# Early post-treatment choroidal thickness to alert sunset glow fundus in patients with Vogt-Koyanagi-Harada disease treated with systemic corticosteroids

**DOI:** 10.1371/journal.pone.0172612

**Published:** 2017-02-27

**Authors:** Kiriko Hirooka, Wataru Saito, Kenichi Namba, Kazuomi Mizuuchi, Daiju Iwata, Yuki Hashimoto, Susumu Ishida

**Affiliations:** 1 Department of Ophthalmology, Hokkaido University Graduate School of Medicine, Sapporo, Japan; 2 Kaimeido Eye and Dental Clinic, Sapporo, Japan; University of Birmingham, UNITED KINGDOM

## Abstract

**Purpose:**

To determine if early post-treatment central choroidal thickness (CCT) changes can predict sunset glow fundus (SGF) development in patients with Vogt-Koyanagi-Harada (VKH) disease treated using systemic corticosteroids.

**Methods:**

This retrospective case series included 39 eyes of 21 treatment-naïve patients with acute VKH disease who could be followed up for more than 12 months after systemic corticosteroid therapy. The eyes were divided into two groups according to whether SGF was present or absent at 12 months (9 eyes of 5 patients versus 30 eyes of 16 patients, respectively). Using enhanced depth imaging optical coherence tomography, CCT values were measured before treatment, then at 1 week and 1 and 3 months after treatment in both groups and compared between the two groups.

**Results:**

Development of SGF was found 4–11 months after treatment. Mean post-treatment CCT decreased significantly at all examinations compared with baseline in both groups, along with resolution of serous retinal detachment. One week after treatment, mean CCT was significantly higher in eyes with SGF than in those without (*P* = 0.024). SGF was present at 12 months in 9 of 22 eyes with CCT values > 410 μm at 1 week after starting treatment, in contrast with none of 17 eyes with CCT ≤ 410 μm at this time (*P* = 0.003).

**Conclusions:**

The current study suggested the potential validity of early post-treatment CCT as a feasible index to alert future progression to SGF in patients with VKH disease treated using systemic corticosteroids.

## Introduction

Vogt-Koyanagi-Harada (VKH) disease is a multi-system disorder that is considered to stem from autoimmunity against melanocytes [1]. In the eye, the disease presents as acute bilateral granulomatous panuveitis, which responds to systemic corticosteroid therapy and had a generally good visual prognosis [2]. However, persistent ocular inflammation seen in chronic recurrent VKH disease frequently leads to vision-threatening ocular complications such as cataract, glaucoma and choroidal neovascularization [3].

Sunset glow fundus (SGF) is a funduscopic finding characterized by orange-red discoloration due to depigmentation of the inflamed choroid [4], and appears in approximately 60–70% of patients with VKH disease at the convalescent stage [5]. Patients with chronic ocular inflammation lasting more than 6 months were found to be more likely to develop SGF and to have poor final vision than those rescued from ocular inflammation within 6 months [6]. Therefore, patients with SGF are suspected to be prone to ocular complications and functional loss as well as non-ocular adverse events secondary to prolonged administration of systemic corticosteroids.

From an anatomic point of view, appearance of SGF has been associated with thinning of the choroid [7, 8] and development of peripapillary atrophy [8] during the convalescent phase of VKH disease, suggesting inflammation-related destruction or loss of chorioretinal tissues as the underlying pathogenesis of SGF [8]. Taken together, to predict the onset of SGF at an earlier stage may theoretically be advantageous in reducing the frequency of ocular and systemic complications in patients with VKH disease by boosting treatments in advance.

Peripapillary atrophy associated with VKH disease was observed at 12 months in eyes with central choroidal thickness (CCT) > 550 μm at 1 week after initiating treatment, but not in any eyes with CCT ≤ 550 μm at this time [9], suggesting a threshold of CCT > 550 μm for predicting the development of peripapillary atrophy. We and others have demonstrated that CCT increases not only during the acute stage of VKH disease but also in association with recurrence of anterior and posterior segment inflammation [7, 9–14], suggesting CCT as a useful marker for clinical evaluation of choroiditis during the chronic course of VKH disease. The present study investigated the utility of CCT to predict development of SGF in patients with acute VKH disease by comparing CCT measurements soon after starting systemic corticosteroid therapy between patients who did and did not develop SGF within 12 months.

## Materials and methods

### Patients and diagnosis

This retrospective case series included 39 eyes from 21 consecutive patients with treatment-naïve acute VKH disease who visited the Intraocular Inflammation Clinic at Hokkaido University Hospital from July 2012 to November 2015 and were followed up for more than 12 months after the start of systemic corticosteroid therapy. VKH disease was diagnosed according to the criteria of Sugiura [15] and the VKH Disease Committee [16]. None of the patients had any medical or ocular history at the initial visit. CCT changes were assessed by enhanced depth imaging optical coherence tomography (EDI-OCT) for up to 3 months after treatment. The EDI-OCT images used in this study included ones that have been published earlier [12, 13]. Exclusion criteria were suspicion of recurrent VKH disease with an SGF appearance at the initial visit and absence of any of the regular EDI-OCT recordings during the 3-month period. The study was approved by the ethics committee of Hokkaido University Hospital (#015–0162) and followed the tenets of the Declaration of Helsinki. Written informed consent whose procedure was approved by the ethics committee was obtained from all subjects for chart review after the nature and possible consequences of the study had been explained.

The eyes were divided into two groups according to the presence or absence of SGF at 12 months (i.e., an SGF group and a non-SGF group). Using patients’ medical charts describing ocular findings at approximately monthly intervals, a single investigator (KM) routinely determined the onset of SGF based on appearance of the funduscopically orange-red discoloration that had not been present at the visit 1 month earlier. The presence of SGF in each eye was then reconfirmed by one of another two experts (KN or DI) by checking the color fundus pictures.

### Ophthalmologic examinations

At their initial visit, patients underwent comprehensive ophthalmic examinations, including decimal best-corrected visual acuity (BCVA), indirect ophthalmoscopy, color fundus photography, fluorescein angiography, indocyanine green angiography, spectral-domain optical coherence tomography (SD-OCT), and EDI-OCT (RS-3000 Advance; Nidek, Gamagori, Japan). During follow-up, EDI-OCT measurements were performed every week for up to 4 weeks after treatment and at monthly intervals thereafter.

### Treatment

Patients received either of two corticosteroid regimens of large-dose or pulse therapy according to the timing of their first visit. In 9 patients visiting before April 2014 (3 cases with SGF and 6 cases without SGF), intravenous prednisolone was administered and tapered from 200 mg/day (i.e., high-dose therapy) as described previously [12]. In the remaining 12 patients visiting from April 2014 onwards (2 cases with SGF and 10 cases without SGF), intravenous methylprednisolone was administered at 1,000 mg/day for 3 consecutive days followed by a taper of oral prednisolone (i.e., pulse therapy) as described previously [13].

Oral prednisolone was temporarily increased or restarted if there was an anterior or posterior recurrence of VKH disease. Oral cyclosporine was also administered at and after the second recurrence concurrently with oral prednisolone. In the present study, recurrences were defined as the recurrence of anterior chamber cells and/or posterior segment lesions (e.g., serous retinal detachment [SRD], choroidal folds) detected by slit-lamp biomicroscopy, indirect ophthalmoscopy, and SD-OCT, but not with indocyanine green angiography or EDI-OCT, as based on criteria reported elsewhere [14, 17].

### EDI-OCT

At the initial visit, patients underwent SD-OCT using conventional cross-sectional retinal B-scans of 5 × 5 lines (a scan length of 6.0 mm) passing through the macula both horizontally and vertically. The height of SRD scanned at the top was manually measured from the inner border of the retinal pigment epithelium to the outer border of the detached sensory retina. Additionally, EDI-OCT was performed to determine CCT by manually measuring the distance beneath the fovea from the outer border of the retinal pigment epithelium to the inner border of the sclera. YH and KH independently evaluated OCT images in a masked fashion, not knowing any subject’s clinical information. CCT reaching > 800 μm was defined as 800 μm, because the inner scleral border could not be visualized with EDI-OCT. Differences in average CCT values were statistically compared between the SGF and non-SGF groups at each stage and between pre-treatment and post-treatment stages in each group.

### Statistical analysis

Values were expressed as the mean ± standard deviation. Decimal BCVA was converted to the logarithm of minimal angle of resolution (logMAR). The Friedman test and Wilcoxon’s signed-rank test were used to compare sequential changes in logMAR BCVA and CCT in each group. The Mann-Whitney *U* test or Fisher’s Exact test was used to compare various parameters between the SGF and non-SGF groups. For all tests, *P* < 0.05 was considered to be statistically significant.

## Results

### Pre-treatment parameters

**Fig 1:**
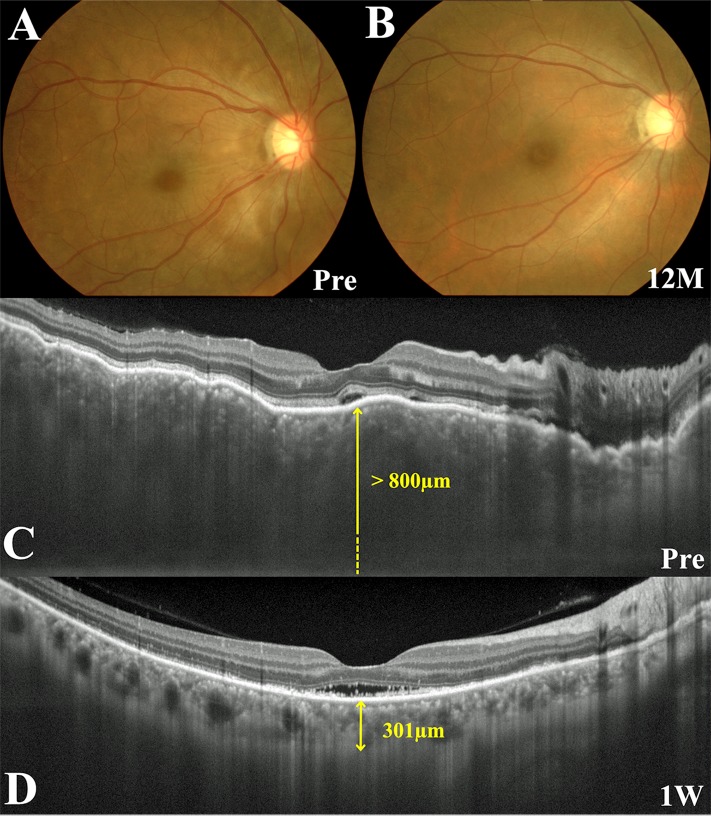
Images of the right eye in a patient with Vogt-Koyanagi-Harada disease who did not develop sunset glow fundus (SGF) during follow-up (Case 15). (A, B) Fundus photographs show serous retinal detachment (SRD) extending from the peripapillary area to the posterior pole, choroidal folds, and optic disc swelling before treatment (A) and complete resolution of SRD without onset of SGF at 12 months after the start of systemic corticosteroid therapy (B). (C, D) Horizontal images through the fovea on enhanced depth imaging optical coherence tomography. A pretreatment image demonstrates SRD extending from the optic disc to the macula, bumpy undulation of the retinal pigment epithelium, and marked choroidal thickening. Central choroidal thickness (CCT) was > 800 μm (C). One week after initiation of systemic corticosteroid therapy, the SRD in the vicinity of the disc disappeared and the CCT decreased to 301.0 μm (D).

**Fig 2:**
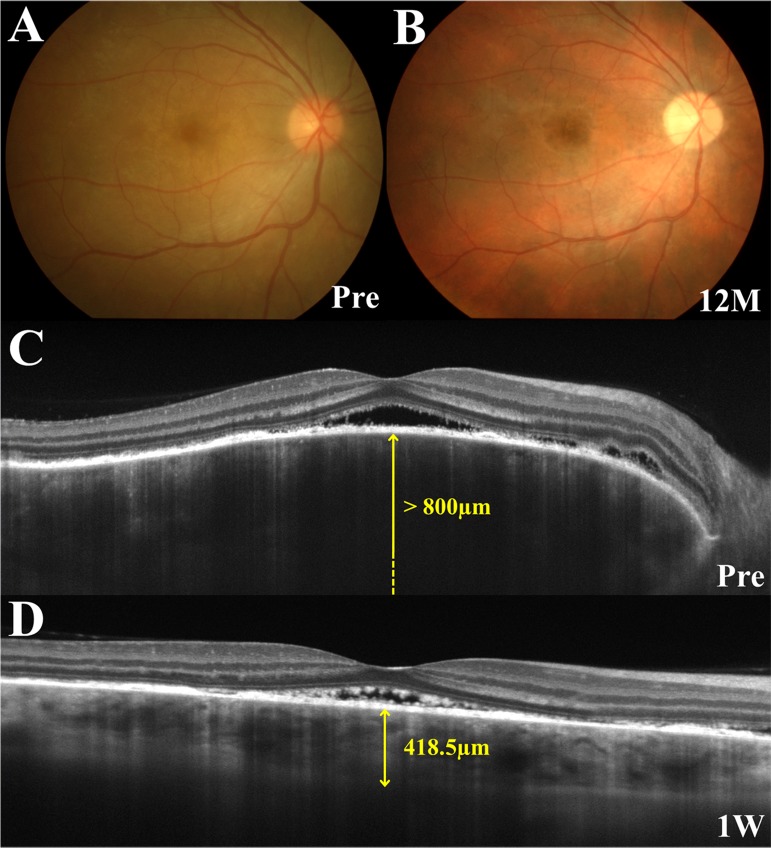
Images of the right eye in a patient with Vogt-Koyanagi-Harada disease who developed sunset glow fundus (SGF) during follow-up (Case 4). (A, B) Fundus photographs show serous retinal detachment (SRD) extending from the disc to the macula and optic disc swelling before treatment (A) and the presence of SGF with disappearance of SRD at 12 months (B). (C, D) Horizontal images through the fovea on enhanced depth imaging optical coherence tomography. An image taken before treatment demonstrates SRD at the posterior pole and marked choroidal thickening. Central choroidal thickness (CCT) was > 800 μm (C). One week after start of systemic corticosteroids, the area of SRD reduced and the CCT also decreased to 418.5 μm (D).

Baseline patient characteristics are summarized in Table 1. Based on the presence of SGF at 12 months, 9 eyes of 5 patients were classified into the SGF group and the remaining 30 eyes of 16 patients into the non-SGF group. None of the eyes enrolled in this study developed SGF after 12 months. Before treatment, SRD was detected at the macular area in 7 of 9 eyes with SGF and 20 of 30 eyes without SGF (Figs 1A, 1C, 2A and 2C). At baseline, no statistically significant difference was detected between the two groups in any of the following parameters examined: age, sex, refractive error, logMAR BCVA, height of SRD, rate of cerebrospinal fluid pleocytosis, and duration from onset to treatment.

**Table 1:** Baseline Characteristics of Patients with VKH Disease with or without SGF.

	SGF (9 eyes of 5 cases)	Non-SGF (30 eyes of 16 cases)	*P*-value
age (years)	38.4±24.8	40.6±12.6	0.54^$^
Sex (male:female)	2:3	5:11	1.000^*#*^
Refractive error (diopters)	-1.7±2.2	-0.9±2.7	0.39^$^
LogMAR BCVA	0.1±0.2	0.2±0.4	0.60^$^
Height of SRD (μm)	203±286.9	259.8±341.3	0.73^$^
Pleocytosis (+:—)	5:0	14:2	1.000^*#*^
Duration from onset to treatment (days)	14.2±3.5	24.6±23.9	0.65^$^

VKH = Vogt-Koyanagi-Harada; SGF = sunset glow fundus; BCVA = best-corrected visual acuity; SRD = serous retinal detachment

# Fisher's exact test

$ Mann-Whitney U test

### Post-treatment parameters

SRD disappeared within 3 months after the start of treatment in all eyes. The clinical parameters compared post treatment between the SGF (Cases 1–5) and non-SGF (Cases 6–21) groups are summarized in Table 2. First, there was no significant difference in the mean follow-up duration between the two groups, in accordance with the relatively early onset of SGF ranging 4 from 11 months (mean 6.3±2.6 months) in the SGF group. This is consistent with the finding of no difference between the SGF and non-SGF groups with regard to choice of treatment regimen according to the period during which patients visited the clinic (i.e., large dose, earlier; pulse, later). As expected, the number of recurrences within 12 months was significantly much higher in the SGF group than in the non-SGF group.

**Table 2:** Post-Treatment Course of Patients with VKH disease with or without SGF.

Case	Duration to SGF onset (M) (R/L)	Follow-up period (M)	Initial treatment (large dose:pulse)	Duration to recurrence (M) (R/L)	Number of recurrence (R/L)	Post-treatment ocular complications (R/L) (+:—)	Final logMAR BCVA (R/L)
1	11/11	33	Large dose	6/6	1/1	Cataract/Cataract	0.00/0.22
2	6/6	30	Pulse	1/1	3/3	Cataract/Cataract	0.00/-0.08
3	5/5	25	Large dose	6/6	1/1	None/None	-0.18/-0.18
4	4/4	48	Large dose	‐/‐	0/0	None/None	-0.08/-0.08
5	‐/5	51	Pulse	‐/0.75	‐/3	‐/Glaucoma	‐/0.00
SGF	6.3±2.6	37.4±10.2	3:2	3.8±2.5	1.4±1.2	5:4	0.0±0.1
6	‐/‐	36	Large dose	‐/‐	0/0	None/None	-0.18/-0.18
7	‐/‐	25	Pulse	‐/‐	0/0	None/None	-0.18/-0.18
8	‐/‐	33	Large dose	‐/‐	0/‐	None/‐	-0.18/‐
9	‐/‐	35	Large dose	‐/‐	0/0	Cataract/Cataract	-0.18/-0.18
10	‐/‐	37	Large dose	‐/‐	0/0	Cataract/Cataract	-0.18/-0.08
11	‐/‐	26	Pulse	‐/‐	0/0	None/None	0.05/-0.18
12	‐/‐	25	Pulse	‐/‐	0/0	Glaucoma/Glaucoma	-0.18/-0.08
13	‐/‐	34	Large dose	‐/‐	0/0	None/None	-0.08/-0.08
14	‐/‐	27	Large dose	‐/‐	0/0	Cataract/Cataract	-0.18/-0.08
15	‐/‐	25	Pulse	4/4	1/1	None/None	0.00/0.00
16	‐/‐	28	Pulse	‐/1.5	‐/3	‐/None	‐/-0.18
17	‐/‐	13	Pulse	5/5	1/1	None/None	0.00/0.00
18	‐/‐	24	Pulse	‐/2.5	0/1	None/None	-0.08/-0.08
19	‐/‐	24	Pulse	‐/‐	0/0	None/None	0.00/-0.08
20	‐/‐	23	Pulse	‐/‐	0/0	None/None	-0.08/-0.08
21	‐/‐	24	Pulse	‐/‐	0/0	None/None	-0.08/-0.08
Non-SGF		27.4±6.0	6:10	3.7±1.3	0.3±0.6	8:22	-0.1±0.1
*P*-value	N.A.	0.081^$^	0.61^#^	N.A.	**0.0008^$^	0.13^#^	0.13^$^

BCVA = best-corrected visual acuity; N.A. = not applicable

# Fisher's exact test

$Mann-Whitney U test.

** *P*<0.01

* *P*<0.05

### Visual prognosis

Of 39 eyes examined, we documented mild to moderate cataract in 10 eyes and drug-controllable glaucoma in 3 eyes after treatment, none of which required surgery during follow-up. There was no difference in the positive-negative ratio of these ocular complications between the SGF and non-SGF groups (Table 2). The mean logMAR BCVA values at baseline, 3 months after treatment, and at the final visit were 0.1 ± 0.2, 0.1 ± 0.3, and 0.0 ± 0.1 in the SGF group and 0.2 ± 0.4, -0.1 ± 0.1, and -0.1 ± 0.1 in the non-SGF group, indicating significant visual improvements over time from baseline to the final visit in the non-SGF group but not in the SGF group (non-SGF, *P* = 0.0001; SGF, *P* = 0.07; Friedman test). The final BCVA showed no significant difference between the two groups (Table 2).

### CCT changes

**Fig 3:**
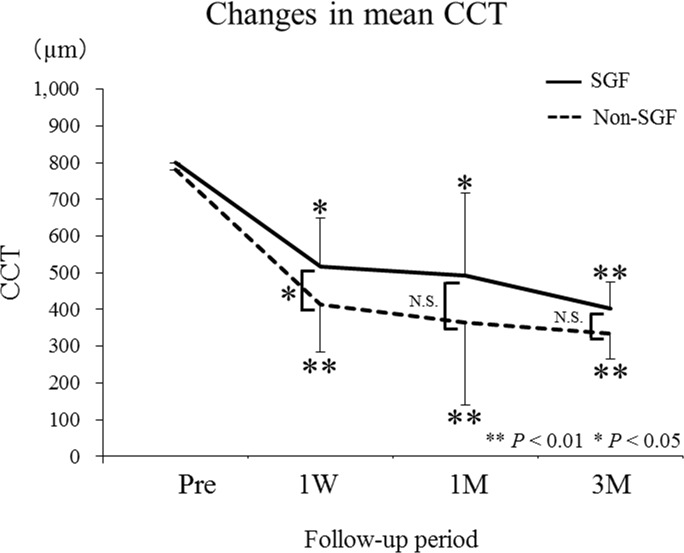
Changes in mean central choroidal thickness (CCT) in the sunset glow fundus (SGF) and non-SGF groups before and after systemic corticosteroid therapy. The mean post-treatment CCT decreased significantly at all examinations in both groups when compared with baseline. Note that the mean CCT at 1 week after starting treatment was significantly higher in the SGF group (518.2 ± 131.6 μm) than in the non-SGF group (414.8 ± 141.6 μm).

The mean CCT values pretreatment and at 1 week, and 1 and 3 months after treatment were 800.0 ± 0.0, 518.2 ± 131.6, 492.8 ± 224.5, and 403.3 ± 70.2 μm in the SGF group, and 780.5 ± 73.7, 414.8 ± 141.6, 364.0 ± 107.9, and 334.5 ± 90.1 μm in the non-SGF group (Fig 3), indicating significant changes during the 3 months of follow-up (SGF, *P* = 0.005; non-SGF, *P* < 0.001; Friedman test). In both groups, the mean post-treatment CCT values decreased significantly at all examinations when compared with baseline (SGF, *P* = 0.01, 0.03, and 0.008; non-SGF, *P* < 0.001 for all; Wilcoxon signed rank test). One week after treatment, the mean CCT was significantly higher in the SGF group than in the non-SGF group (*P* = 0.024). This pattern continued but waned at 1 and 3 months, showing no statistically significant difference between the groups.

**Fig 4:**
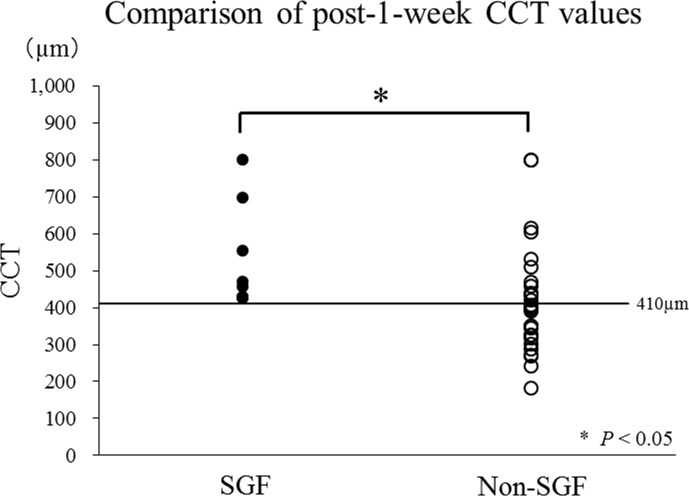
Comparison of central choroidal thickness (CCT) between the sunset glow fundus (SGF) and non-SGF groups at 1 week after starting treatment. There was a significant difference in the thick:thin ratio with a cut-off value of 410 μm for CCT between the SGF (9:0 eyes) and non-SGF (13:17 eyes) groups at 1 week after starting treatment (*P* = 0.003).

Since the lowest value for CCT in the SGF group at 1 week was 416 μm, an arbitrary cut-off value of 410 μm was set for further statistical comparison (Fig 4). SGF developed in 9 of 22 eyes with CCT > 410 μm at 1 week, in contrast with none in 17 eyes with CCT ≤ 410 μm, indicating a significant difference in the thick:thin ratio (> 410 μm:≤ 410 μm) of post-treatment CCT between the SGF (9:0 eyes) and non-SGF (13:17 eyes) groups (*P* = 0.003, Fisher’s Exact test). The sensitivity and specificity values for post-1-week CCT > 410 μm as a predictor of SGF were 100% (9 of 9 eyes) and 56.7% (17 of 30 eyes), respectively.

## Discussion

The present study compared early post-treatment CCT values between eyes with and without SGF present 12 months after initiating systemic corticosteroid therapy to predict later development of SGF in treatment-naïve patients with VKH disease. The mean CCT values in both groups gradually decreased after systemic corticosteroid treatment and the values at all examinations after the start of treatment were significantly reduced when compared with the pretreatment values. However, the CCT at 1 week in eyes with SGF was significantly greater than in eyes without SGF and there was a significant difference in the frequency of eyes divided into CCT of > 410 μm or ≤ 410 μm at 1 week between the SGF and non-SGF groups, suggesting that the cut-off value at 1 week for CCT that later developed into SGF was 410 μm. To the best of our knowledge, this study is the first to show a relationship between appearance of SGF and the early post-treatment CCT response in VKH disease.

Previous studies have reported that eyes with SGF showed choroidal thinning in the convalescent phase of VKH disease when compared with age-matched normative eyes and eyes with VKH disease developing no SGF [7, 8]. However, in the present study, CCT value at 1 week after initiating systemic corticosteroid therapy in eyes that developed SGF was significantly higher than in eyes without SGF and the tendency continued during the 3-month follow-up period. It is known that appearance of SGF is associated with persistent ocular inflammation continuing for more than 6 months [6]. In the current data, eyes with SGF had more recurrences than those without SGF and showed no significant improvement of BCVA during follow-up. Therefore, our results suggest that the severity of inflammation in the choroid continues to be more intense in eyes with SGF during the early post-treatment period. A possible explanation for this is that eyes destined to develop SGF in the future might have a poorer response to systemic corticosteroid therapy or initial development of more severe choroiditis. However, it would be difficult to determine whether SGF is caused by either of these mechanisms because accurate measurements of choroidal thickness in the initial stage of this disease are difficult to obtain using the currently available techniques, including swept-source OCT [7]. Further studies are needed to compare baseline choroidal parameters other than thickness between eyes with or without SGF.

In this study, we have shown that CCT in eyes with SGF was significantly greater than in eyes without SGF when measured 1 week after starting treatment. This significant difference would not result from the temporary increase in CCT caused by recurrence of ocular inflammation, because no eye in either group had a recurrence during the week after starting treatment. Therefore, the narrow window of time detected in the present study would be of great clinical importance as an early alert for later onset of SGF during the convalescent phase. Moreover, our current data showed that the predictive cut-off value for risk of future progression to SGF was 410 μm with a significantly (*P* = 0.003) different thick:thin ratio between the SGF (9:0 eyes) and non-SGF (13:17 eyes) groups. Importantly, the statistical significance at 410 μm was maintained even if one eye per patient was analyzed to avoid any systemic confounders, showing a less but still significant (*P* = 0.038) difference in the thick:thin ratio between the SGF (5:0 eyes) and non-SGF (6:9 eyes) groups, together with sensitivity and specificity values of 100% (5 of 5 eyes) and 60% (9 of 15 eyes), respectively (data not shown). Close attention should be paid to these high-risk patients with CCT > 410 μm at 1 week to allow timely intervention by boosting treatment (e.g., increasing the dose of corticosteroids, using additional immunosuppressants) to improve their long-term outcomes.

This study has some limitations, including a retrospective design and small sample size. Next, a deviation in the number of patients between the two groups may have influenced the results. Then, patients in both groups received treatments of two regimens according to the timing of patients’ first visit. Mixture of two regimens may have influenced the results, although there was no significant difference in the ratios of the two regimens between both groups. In the future, further studies are needed to compare CCT between two groups receiving the same regimen.

## Conclusions

In patients with acute VKH disease receiving systemic corticosteroid therapy, the mean CCT at 1 week after the start of treatment in eyes with SGF was significantly greater than in eyes without SGF and the cut-off CCT value at 1 week to alert future progression to SGF was 410 μm. These results suggest that the early post-treatment CCT may be a feasible predictor of later development of SGF during the course of VKH disease. Prospective multicenter interventional studies with larger sample sizes are now needed to confirm our findings.
